# A practical approach for a patient-tailored dose protocol in coronary CT angiography using prospective ECG triggering

**DOI:** 10.1007/s10554-015-0802-z

**Published:** 2015-11-12

**Authors:** J. D. van Dijk, E. D. Huizing, P. L. Jager, J. P. Ottervanger, S. Knollema, C. H. Slump, J. A. van Dalen

**Affiliations:** Department of Nuclear Medicine, Isala Hospital, PO Box 10400, 8000 GK Zwolle, The Netherlands; Department of Cardiology, Isala Hospital, Zwolle, The Netherlands; Department of Medical Physics, Isala Hospital, Zwolle, The Netherlands; MIRA: Institute for Biomedical Technology and Technical Medicine, University of Twente, Enschede, The Netherlands

**Keywords:** Radiation dosage, Body size, Cardiac angiography, Computed X-ray tomography, Cardiac imaging techniques, Coronary artery disease

## Abstract

To derive and validate a practical patient-specific dose protocol to obtain an image quality, expressed by the image noise, independent of patients’ size and a better radiation dose justification in coronary CT angiography (CCTA) using prospective ECG triggering. 43 patients underwent clinically indicated CCTA. The image noise, defined as the standard deviation of pixel attenuation values in a homogeneous region in the liver, was determined in all scans. Subsequently, this noise was normalized to the radiation exposure. Next, three patient-specific parameters, body weight, body mass index and mass per length (MPL), were tested for the best correlation with normalized image noise. From these data, a new dose protocol to provide a less variable image noise was derived and subsequently validated in 84 new patients. The normalized image noise increased for heavier patients for all patients’ specific parameters (*p* < 0.001). MPL correlated best with the normalized image noise and was selected for dose protocol optimization. This new protocol resulted in image noise levels independent of patients’ MPL (*p* = 0.28). A practical method to obtain CCTA images with noise levels independent of patients’ MPL was derived and validated. It results in a less variable image quality and better radiation exposure justification and can also be used for CT scanners from other vendors.

## Introduction

For patients with suspected stable coronary artery disease it is recommended to perform non-invasive testing prior to invasive coronary angiography [1]. In patients with a low to intermediate pre-test probability for coronary artery disease, use of computed tomography coronary angiography (CCTA) is advised [1]. To reduce the high radiation burden associated with CCTA, prospective ECG-triggering was introduced [2]. This technique activates the X-ray tube only in the end-diastolic phase rather than throughout the cardiac cycle, resulting in dose reductions up to 90 % [3, 4]. However, prospective ECG-triggering cannot be used in combination with automatic anatomy-based tube-current modulation which corrects for the varying patients’ size [4]. This correction ensures a less variable image quality and, hence, sufficient diagnostic CCTA image quality with a minimum radiation exposure. Only few CT scanners have the possibility to automatically adjust the tube settings based on a preceding image, as alternative to anatomy-based tube-current modulation, to correct for the varying patients’ size. Hence, manual adjustment prior to imaging is required in most scanners [3–6].

Multiple CCTA protocols are proposed to adapt for varying patient size to obtain a less variable image quality [7–14]. Most of them modify tube settings based on image noise found in a preceding scan, for example a bolus scan. Although these protocols result in a less variable image noise, it is cumbersome to implement them in clinical practice. Consequently, most institutes nowadays use protocols that are empirically adjusted, using body mass index (BMI) or weight, not necessarily resulting in a constant level of image noise. However, image noise is closely related to image quality [7–11]. In particular, a constant image noise will result in a less variable image quality. But only few studies describe clinical applicable dose protocols for specific CT imaging configurations that result in less variable image noise [10–13]. Moreover, a general method to derive these protocols for different CT settings or scanners is lacking. Therefore, these methods cannot be enrolled at other centers without additional efforts. Hence, the aim of our study was to demonstrate how to derive and validate a practical patient-tailored CCTA imaging protocol using prospective ECG-triggering in order to obtain an image quality, expressed by the image noise, independent of patient’s size and thereby providing a better radiation dose justification.

## Materials and methods

### Study population

All 129 retrospectively included patients underwent clinically indicated prospective ECG-triggered CCTA (Discovery NM 570c, GE Healthcare). The first 45 patients were consecutively included to derive a patient-tailored dose protocol (further referred to as group A). For the validation part of this study, 84 additional patients were included (further referred to as group B), of which 43 patients were included consecutively. To obtain a population in the full expected range of body mass per body length (MPL) to demonstrate the validity of the protocol an additional 41 patients were included to obtain at least 10 patients in each of the following MPL categories: <40, 40–45, 46–50, 51–55 and >55 kg/m. These patients were consecutively included for each category. Multiple patient-specific parameters and coronary artery disease risk factors were collected for all patients prior to scanning. As this study was set up in a retrospective manner, no approval by the medical ethics committee was required. All patients provided written informed consent for the use of their data for research purposes.

### Patient preparation and image acquisition

Patients were instructed to remain fasting for 3 h prior to acquisition. Patients with heart rates between 49 and 59 or >59 beats per minute were requested to take 50 or 100 mg metoprolol orally, respectively, 1 h prior to acquisition. Diazepam (10 mg) was administered when clinically indicated to calm the patients for additional heart rate reduction.

Patients were scanned in supine position, with arms placed above their head. A scout image (120 kV, 10 mA) was acquired prior to the bolus acquisition to determine the scan field. Bolus delay was determined by making 10 consecutive acquisitions in 20 s (120 kV, 60 mA). Next, patients were administered two puffs (2 × 0.4 mg) of nitroglycerine sublingual, unless contraindicated.

All CT-scans were prospectively ECG-triggered at 75 % of the RR interval and were acquired using the following parameters: collimation 64 × 0.625 mm, rotation time of 0.35 s and a tube voltage depending contrast flow of 4 ml/s at 100 kV, 5 ml/s at 120 kV, and 6 ml/s at 140 kV (Optiray^tm^, Mallinckrodt). The standard applied BMI depending protocol in our institution, as applied in group A, is shown in Table 1. The CT scans were reconstructed using filtered back projection with a slice thickness of 0.625 mm, 512 × 512 matrix and a pixel size of 0.35 mm (Xeleris software, GE Healthcare).

**Table 1:** The applied BMI depending dose protocol for patients in group A including tube settings and estimated radiation dose

BMI (kg/m^2^)	Tube current (mA)	Tube voltage (kV)	CTDI (mGy)	Effective dose (mSv)
<17	360	100	4.4	1.0
17–19	400	100	4.9	1.1
19–21	415	100	5.1	1.2
21–23	440	100	5.3	1.2
23–24	320	120	6.4	1.5
25–29	360	120	7.2	1.7
30–35	465	120	9.3	2.2
>35	410	135	10.8	2.5

### Deriving a patient-specific CCTA protocol

**Fig. 1:**
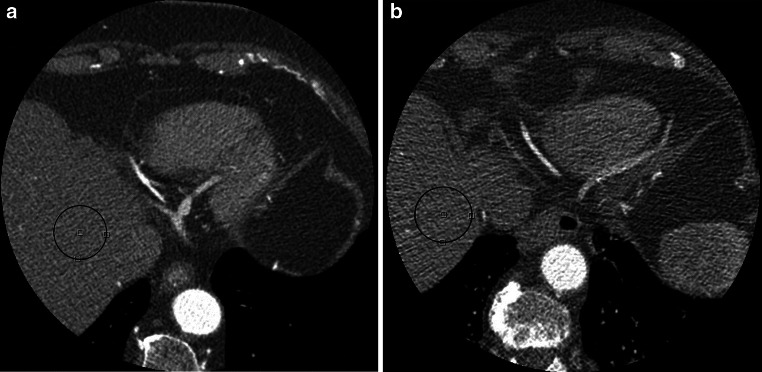
Example of axial slices of two CCTA scans, including the regions of interests, demonstrating the increase in image noise and decrease in image quality in heavier patients. Both scans are from female patients. **a** A lean patient of 69 kg, BMI 24.4 kg/m^2^ and MPL 41.1 kg/m and **b** a more obese patient of 89 kg, BMI 39.6 kg/m^2^ and MPL 59.3 kg/m. Images were acquired using the same tube voltage of 120 kV and tube current of 400 mA. The measured image noise for the lean patient is 47 HU and for the more obese patient 87 HU

The image noise, defined as the standard deviation of pixel attenuation values in a visually homogeneous region of interest (ROI), was measured in the most cranial part of the liver parenchyma in each scan, as illustrated in Fig. 1. Next, image noise was fitted to multiple patient-specific parameters (P) which were considered easy applicable in daily use; body weight, BMI and MPL, to determine a possible increase in image noise for heavier patients (see Table 1).

To determine the relation between image noise and patients’ size for a fixed radiation dose, the measured image noise was normalized to the squared root of the applied computed tomography dose index (CTDI) expressed in mGy. This was based on the formula previously used by Menke et al. [15]:

1 $${\text{CTDI}} \cdot \upsigma^{2} \propto {\text{e}}^{{\upmu \cdot {\text{d}}}}$$

Here σ is the measured image noise, µ the mean attenuation coefficient of the region at a defined tube voltage (cm^−1^) and d the axial diameter of the patient (cm). Subsequently, for each patient a normalized value of image noise (σ_norm_) was determined using:

2 $$\upsigma_{\text{norm}} = \upsigma \cdot \surd {\text{CTDI}}$$

Next, the relations between the σ_norm_ and multiple patient-specific parameters (P) were investigated to find the parameter best explaining the relation between σ and P. Therefore, σ_norm_ was fitted using a linear function (σ_fit_):

3 $$\upsigma_{\text{fit}} = {\text{a}} \cdot {\text{P}} + {\text{b}}$$

Here, a and b are fit parameters.

### Patient-specific CTDI

When combining Eqs. 2 and 3, with σ_norm_ described by the linear function σ_fit_, we obtained a new CTDI (CTDI_apply_):

4 $${\text{CTDI}}_{\text{apply}} = \left( {\frac{{\upsigma_{\text{fit}} }}{{\upsigma_{\text{C}} }}} \right)^{2} = \left( {\frac{{{\text{a}} \cdot {\text{P}} + {\text{b}}}}{{\upsigma_{\text{C}} }}} \right)^{2}$$

Here σ_C_ is the desired constant image noise, which was set equal to the average image noise measured in all patient scans in this study. Ideally, the noise becomes independent of the patient examined when applying the new CTDI using the appropriate tube settings (kV and mA). The choice of tube voltages was based on tube voltage guidelines using weight and BMI; 100 kV below 90 kg or 30 kg/m^2^ corresponding to a MPL of 45 kg/m, 140 kV for severely obese patients (MPL > 60 kg/m) and 120 kV for the remainder of the patients [4]. Next, the tube currents were derived using these tube voltages to obtain CTDI_apply_. Yet due to the maximum tube current achievable on the CT scanner, a higher tube voltage of 120 kV was used for MPLs between 45 and 52.5 kg/m to obtain CTDI_apply_.

To ensure validity of the protocol, it was derived for patients with a body weight between 60 and 130 kg, BMI between 17 and 35 kg/m^2^ or MPL between 35 and 60 kg/m. Patients outside this pre-specified range received the minimal or maximal recommended radiation dose, i.e. a patient with a MPL of 30 kg/m received the dose corresponding to a patient of 35 kg/m. The effective dose was estimated using the mean irradiated body length of 13.7 cm and the thorax conversion factor of 0.017 mSv/mGy/cm [16].

### Validation

The optimized patient-specific CCTA protocol was implemented as a new routine clinical protocol. Next, to examine if the image noise was independent of patients’ size using the new protocol, the best explaining parameter P was correlated to the image noise for patients within the pre-specified range in groups A and B.

### Statistics

All patient characteristics for groups A and B were presented as mean ± standard deviation (sd) and compared using the χ^2^ and unpaired *t* tests using Stata software (StataSE 12.0). To test if the regression coefficients of the σ_fit_ for each patient-specific parameter P differed significantly from zero, implying a significant correlation between σ and P or σ_norm_ and P, *t* tests were performed. Coefficients of determination, R^2^, were determined for all fits and compared using the Hotelling–Williams test. Using the results of R^2^ and the Hotelling–Williams tests, the patient-specific parameter best explaining the σ_norm_ was selected for the validation study.

The level of statistical significance was set to 0.05 for all statistical analyses.

## Results

The baseline characteristics of all included patients are summarized in Table 2.

**Table 2:** Baseline characteristics of all 129 included patients who underwent clinically indicated prospective ECG-triggered CCTA

Characteristic	Group A (n = 45)	Group B (n = 84)	*p* value (χ^2^/*t* test)
Age (years)	60.2 ± 12.2	54.9 ± 12.0	0.02
Male gender (%)	55.6	56.0	0.97
Body weight (kg)	82.1 ± 16.1	85.6 ± 18.4	0.28
BMI (kg/m^2^)	27.3 ± 5.3	27.9 ± 5.6	0.59
MPL (kg/m)	47.3 ± 8.7	48.8 ± 9.9	0.39
CTDI (mGy)	8.2 ± 3.1	9.1 ± 4.2	0.19
DLP (mGy)	110 ± 44	123 ± 53	0.19
Effective dose (mSv)	1.9 ± 0.7	2.1 ± 0.9	0.19
Pulse during scan (BPM)	53.1 ± 7.6	53.6 ± 5.7	0.67

Data are presented as mean ± SD or percentages

### Deriving a patient-specific CCTA protocol

**Fig. 2:**
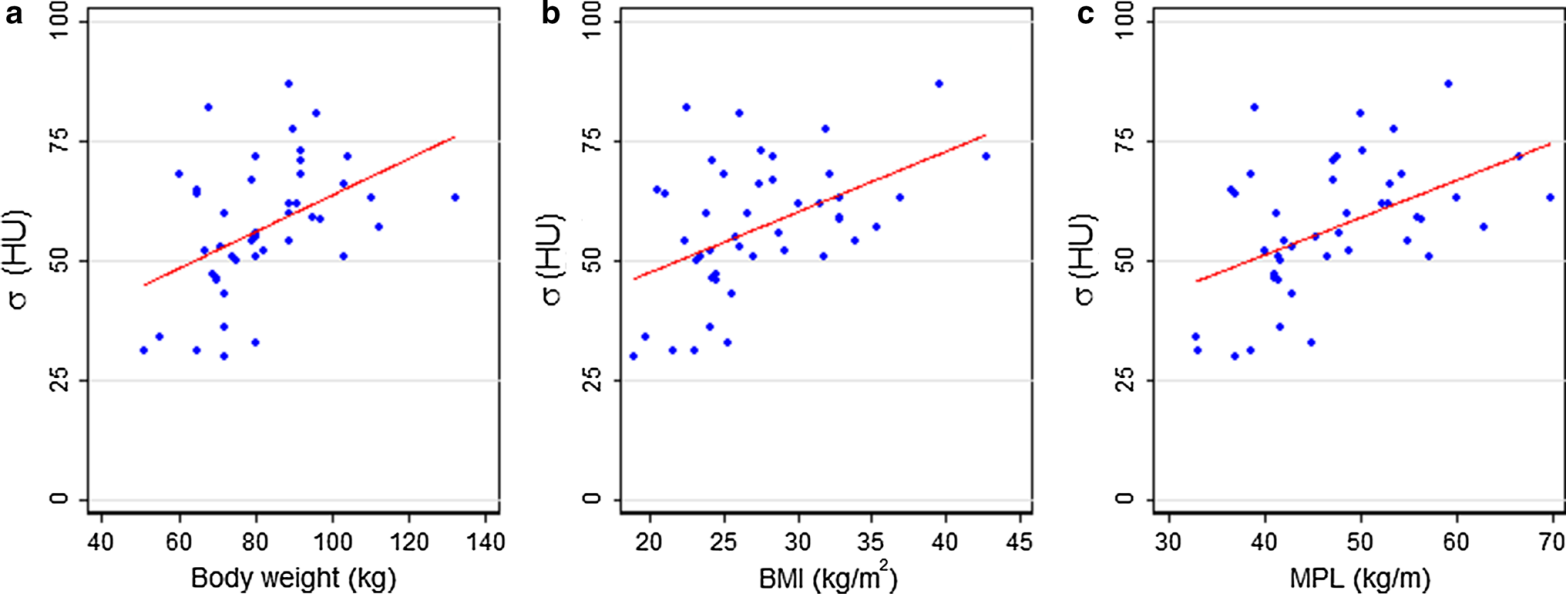
Image noise in the cranial liver parenchyma as a function of three patient-specific parameters in group A; **a** weight, **b** BMI and **c** MPL. *All graphs* show the results of the linear regression fits

The mean measured image noise (σ) and normalized image noise (σ_norm_) in group A was 57 ± 14 HU and 162 ± 52 HU mGy^1/2^, respectively. Despite the applied BMI depending protocol in group A, an increase in image noise was observed for increasing values of all tested patient-specific parameters (*p* ≤ 0.002), as illustrated in Fig. 2.

**Fig. 3:**
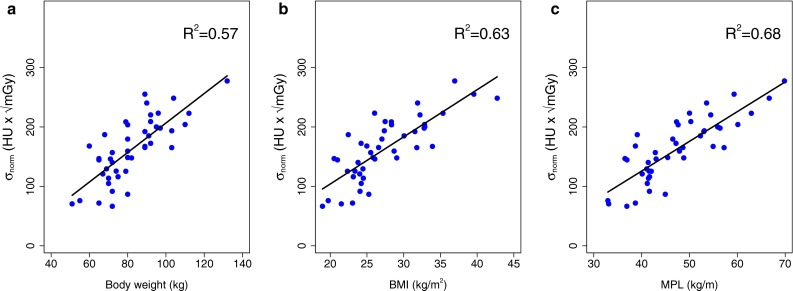
Image noise in the cranial liver parenchyma normalized to the used CTDI as function of three patient-specific parameters; **a** weight, **b**) BMI and **c** MPL. *All graphs* show the results of the linear regression fits. The coefficients of determination for each fit are shown in the *top right corner*

The regression coefficients of the fits describing the σ_norm_ as a function of all three patient-specific parameters were also found to be statistically different from zero (*p* < 0.001), as illustrated in Fig. 3. The calculated fit parameters a and b for all patient-specific parameters are shown in Table 3. MPL had a significantly stronger correlation with the normalized image noise than body weight (*p* = 0.03) but a similar correlation as BMI (*p* = 0.37). Yet based on its R^2^ value, MPL was used in the validation study.

**Table 3:** Results of the fit parameters a and b including the coefficients of determination (R^2^) and the Hotelling–Williams test to compare the correlations

Parameter	*a*	*a* 95 % CI	*b*	*b* 95 % CI	R^2^	Hotelling–Williams test (*p* value)
Body weight (kg)	2.5	1.8–3.1	−42	−96 to 13	0.57	0.03
BMI (kg/m^2^)	7.9	6.1–9.8	−55	−105 to −4	0.63	0.37
MPL (kg/m)	5.0	4.0–6.0	−74	−125 to −24	0.68	–

### Patient-specific CTDI

Using Eq. 4 and the fit parameters a and b, the recommended patient-specific radiation dose using MPL can be described by:

5 $${\text{CTDI}}_{\text{apply}} = \left( {\frac{{5.0 \cdot {\text{MPL}} - 74.2}}{57}} \right)^{2} = \left( {0.088 \cdot {\text{MPL}} - 1.3} \right)^{2}$$

**Fig. 4:**
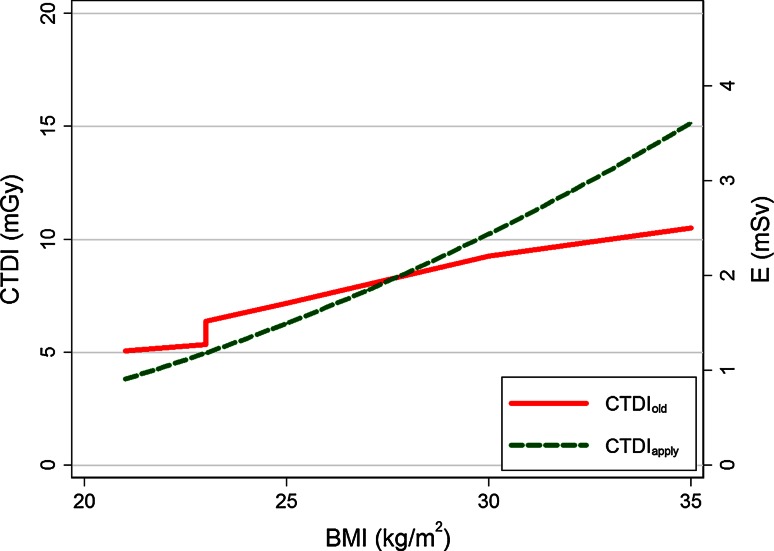
The patient-specific CTDI protocol used for patients in group A (CTDI_old_) and for the new MPL protocol used for patients in group B (CTDI_apply_), converted to a BMI-protocol to allow comparison. The right y-axis shows the corresponding estimated effective dose

The derived radiation dose table describing the proposed CTDI_apply_ is shown in Table 4. In comparison to the protocol as applied in group A, a lower CTDI is recommended for leaner patients and a higher CTDI for more obese patients, as illustrated in Fig. 4.

**Table 4:** Example of a mass per length (MLP) depending dose table, including tube settings and estimated radiation dose, as derived from Eq. 5

MPL (kg/m)	Tube current (mA)	Tube voltage (kV)	CTDI (mGy)	Effective dose (mSv)
<35	265	100	3.1	0.7
37.5	330	100	4.0	0.9
40	410	100	4.9	1.1
42.5	490	100	5.9	1.4
45	580	100	7.0	1.6
47.5	415	120	8.2	1.9
50	480	120	9.5	2.2
52.5	550	120	10.9	2.5
55	620	120	12.4	2.9
57.5	695	120	14.0	3.3
>60	585	140	15.7	3.7

### Validation

**Fig. 5:**
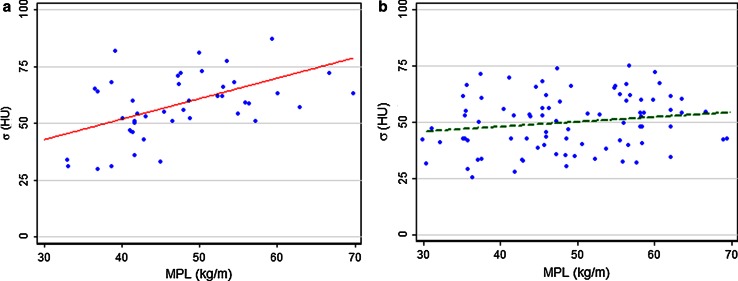
Image noise in the cranial liver parenchyma as a function of MPL for **a** group A and **b** group B including the linear regression fits. While the slope of the fit differed from zero when using a BMI-depending protocol in group A (*p* = 0.007) this was no longer the case after applying the new protocol in group B (*p* = 0.28)

The mean image noise in group B was 50 ± 12 HU and the normalized image noise, σ_norm_, was 147 ± 57 HU mGy^1/2^. Different relations between image noise and MPL were observed for groups A and B, as illustrated in Fig. 5. Whereas the slope of the regression line differed significantly from zero for group A (*p* = 0.007), this was not the case for group B (*p* = 0.28).

## Discussion

The present study demonstrates a method to derive and validate a practical patient-specific prospective ECG-triggered CCTA protocol to overcome the increasing image noise in heavier patients. The protocol is based on the relation between MPL and image noise normalized to the radiation exposure and can also easily be adopted on CT scanners from other vendors, independent of the acquisition and reconstruction settings used. Hence, it provides a useful alternative to CT scanners which can automatically adjust the tube current and voltage based on a preceding scan in combination with prospective ECG-triggering. Applying an MPL dependent protocol resulted in constant image noise levels, independent of patients’ size.

Our findings are in agreement with previous studies proposing patient-specific protocols for prospective ECG-triggered CCTA [7–14, 17]. Most of these studies propose correction protocols based on the attenuation or image noise in preceding scans, for example in a bolus scan. This approach is also used in the newest generation of CT scanners which can automatically adjust the tube current and voltage based on the scout in combination with prospective ECG-triggering. It could result in less variation in image noise than when using a patient-specific parameter as we derived [7–9, 18]. However, our method has several major advantages over the methods which are based on a preceding scan. It can easily be applied on CT scanners from other vendors independent of the acquisition and reconstruction methods used and it requires fewer manual interactions during the scan which shortens the procedure time. Moreover, it does not require a certain radiation exposure of the preceding scans for sufficient noise measurements, lowering the cumulative radiation exposure.

MPL was chosen as the correcting parameter in this study based on its stronger correlation with normalized image noise in comparison to BMI (R^2^ = 0.68 and 0.63, respectively). The choice of MPL may be interpreted as arbitrary. However, when seeing the body morphology as a cylinder, the mass per length provides an estimate of the cross-sectional area of a patient and therefore thickness, intuitively making more sense than dividing the mass by a squared length, like at BMI. In the study by Li et al. [18] they tried to identify the parameter best explaining the image noise in CCTA. They determined that chest circumference at the right coronary artery origin level (R^2^ = 0.60) was the parameter best correcting for the varying patient size. However, they did not test whether this parameter differed significant from BMI (R^2^ = 0.53). Moreover, they did not include any other parameters that can be considered as easily adoptable in clinical practice, such as weight or MPL.

In our study we made several assumptions. First, a more constant image noise level was assumed to result in a better image quality in CCTA. Yet image quality in CCTA also depends on the heart rate, breath holding, iodine enhancement and contrast timing [6, 19, 20]. A qualitative image quality assessment, purely assessing the effect of the obtained constant image noise while excluding the influences of these other parameters, was considered as hardly possible. However, obtaining a less variable quantitative image noise can be seen as an independent and essential first step towards a constant image quality. Second, the image noise was determined in the liver instead of in the thoracic region, as the non-uniform contrast enhancement makes the definition of homogeneous regions of interest difficult [7]. However, the cranial liver parenchyma is typically located on the same axial level of the caudal part of the myocardium and was therefore considered representative for cardiac image noise measurements. Third, the protocol was only derived for patients within a certain body size range (35 kg/m < MPL < 60 kg/m) which might not fully represent the clinical practice. Final, no iterative reconstruction was used. Yet application of iterative reconstruction instead of filtered back projection results in an evenly spread proportional decrease of the image noise which does not compensate for the higher image noise in heavier patients [21]. The method as presented in this study can be used in combination with iterative reconstructions. Moreover, application of iterative reconstructions will allow the use of a lower desired constant image noise (σ_C_) which enables the use of a lower CTDI without compromising image quality [22].

In conclusion, we have derived a MPL dependent CCTA prospective ECG-triggering dose protocol using the proposed method which is also eligible for CT scanners from other vendors. Application of this protocol resulted in an image noise independent of patient’s size. It provided a less variable image quality and better radiation dose justification.

## Abbreviations

CCTA: Coronary computed tomography angiography
CTDI: Computed tomography dose index
MPL: Mass per length (kg/m)
